# Combined Process of Chlorination Roasting and Acid Leaching of Lead and Silver from Lead Cake

**DOI:** 10.3390/ma19010170

**Published:** 2026-01-02

**Authors:** Biserka Lucheva, Peter Iliev, Nadezhda Kazakova

**Affiliations:** Department of Non-Ferrous Metals and Alloys, Faculty of Metallurgy and Materials Science, University of Chemical Technology and Metallurgy, 1756 Sofia, Bulgaria; n_kazakova@uctm.edu

**Keywords:** lead cake, chlorination roasting, acid leaching

## Abstract

This study evaluates an integrated approach for recovering lead and silver from lead cake through chlorination roasting followed by acid leaching. The lead cake originates from sulfuric acid leaching of zinc ferrite residues obtained during the hydrometallurgical processing of zinc calcine. The effects of roasting temperature, lead cake-to-NaCl mass ratio, and roasting duration on metal recovery were systematically examined to determine optimal process conditions. Based on the experimental results, roasting at 550 °C for 1.5 h with a lead cake-to-NaCl mass ratio of 1:3, followed by leaching in 1 M HCl, was selected as a representative and sufficiently effective condition for the combined process. Under these conditions, nearly complete dissolution of Pb and Ag was achieved, reducing their contents in the final solid residue to 0.90% and 0.0027%, respectively. Compared to direct chloride leaching, the combined process provided higher extraction efficiencies (Pb 98.67%, Ag 98.09%) and a lower final residue mass (34% vs. 45%). The roasting step enables the solid-state conversion of PbSO_4_ into highly soluble chloride phases (PbCl_2_ and Pb(OH)Cl), while ZnFe_2_O_4_, Fe_2_O_3_ and SiO_2_ remain stable and form the inert matrix of the residue. Acid leaching at a lower solid-to-liquid ratio (1:10) ensures near-complete dissolution of Pb and Ag, whereas aqueous leaching at a high ratio (1:100) results in incomplete Pb removal. The compliance leaching test (EN 12457-2) confirmed that the residue produced after the optimized two-step treatment meets the EU criteria for inert waste. Overall, the proposed combined process enhances Pb and Ag recovery, minimizes environmental risk, and offers a technically robust and sustainable route for treating lead-containing industrial residues.

## 1. Introduction

Chlorination roasting is a pyrometallurgical process in which metal-containing compounds are converted into their corresponding chlorides in the presence of chlorinating agents. The process relies on differences in the Gibbs free energy of formation and the volatility of metal chlorides, which enables selective separation of target metals even in complex mineral systems. Historically, chlorination roasting was first applied in the extraction of silver and copper, and later extended to magnesium, tin, tungsten, rare earth, and nickel ores. In recent years, it has gained renewed interest for the treatment of metallurgical and electronic wastes, where it serves as an effective route for recovering valuable metals from secondary resources [1].

Depending on the operating temperature, chlorination roasting can be classified as low-temperature, medium-temperature, and high-temperature roasting. Low-temperature chlorination roasting (below 320–400 °C) commonly employs chlorinating agents such as NH_4_Cl or mixtures of NaCl and NH_4_Cl [2,3,4]. In this temperature range, chlorination proceeds through the formation of intermediate chloro-complexes, which subsequently decompose into stable metal chlorides. This approach requires relatively simple equipment and low energy input, making it suitable for oxide and sulfide raw materials of moderate metal content.

Medium-temperature chlorination roasting (400–800 °C) typically uses solid chlorides such as NaCl, CaCl_2_, KCl, and MgCl_2_. Under these conditions, the metal chlorides formed remain in the condensed state, enabling their selective dissolution during subsequent hydrometallurgical processing steps. This method is widely applied for the extraction of metals such as nickel, cobalt, lithium, and rare earth elements from ores and industrial wastes [4,5,6,7,8].

High-temperature chlorination roasting (above 800 °C) leads to the formation of volatile metal chlorides that can be separated via evaporation and condensation. This approach is particularly applicable to metals such as zinc, copper, silver, and gold, as well as complex polymetallic residues [9,10,11].

When lead in waste materials is present in the form of lead sulfate (PbSO_4_), its extraction is typically carried out in chloride media, since PbSO_4_ is poorly soluble and chemically stable in many conventional leaching environments [12,13,14,15,16,17,18,19,20,21].

Although PbSO_4_ can be dissolved under specific conditions, such as in chloride-rich acidic media through complex formation, in nitric acid, in certain organic complexing systems, or in acidic ammonium acetate solutions, these approaches are often associated with strict technological requirements or limited practical applicability. In many cases, effective dissolution requires elevated temperatures, high reagent concentrations, or tailored process conditions developed for specific types of materials. Consequently, despite its conditional solubility, PbSO_4_ remains a challenging phase in the hydrometallurgical processing of complex secondary resources.

Our previous work [22] demonstrated that direct chloride leaching of lead cake using a solution containing 250 g/L NaCl and 1 M HCl achieved high dissolution degrees for Pb (96.79%) and Ag (84.55%). However, the insoluble residue still contained 1.57% Pb, which exceeds typical environmental regulatory thresholds and therefore classifies the residue as hazardous waste. This indicates that direct leaching alone is insufficient to achieve complete lead removal.

A comprehensive literature review indicates that lead recovery from waste materials containing PbSO_4_ is predominantly achieved through direct chloride leaching, sometimes supported by complexing or activating agents. However, studies specifically addressing the application of a combined “chlorination roasting–leaching” process to lead cake or other sulfate-based secondary resources remain limited. This highlights that the mechanisms of solid-state conversion of PbSO_4_ into chloride phases, as well as their influence on the subsequent dissolution behavior, are still insufficiently clarified and require further investigation.

In line with these observations, improving the extractability of Pb from PbSO_4_-containing residues remains a challenging task due to the chemical stability and low solubility of PbSO_4_ in many leaching systems. In this context, thermal activation aimed at phase transformation represents a potential strategy to enhance Pb accessibility. Chlorination roasting, which has been successfully applied in other metallurgical systems to convert metal-bearing compounds into highly soluble chloride phases, was therefore selected in the present study as a pretreatment step for lead cake and evaluated in combination with subsequent hydrometallurgical leaching.

Therefore, the aim of the present study is to investigate the possibility of enhancing the extraction of lead and silver from lead cake through chlorination roasting followed by hydrometallurgical leaching, with the objective of reducing the Pb content in the final residue to levels below the hazardous waste classification threshold.

## 2. Materials and Methods

The initial lead cake was obtained as a result of high-acidity and high-temperature sulfuric acid leaching of zinc ferrite residue [22]. The chemical composition of this material was determined by Inductively Coupled Plasma Optical Emission Spectroscopy (ICP-OES) using a Prodigy spectrometer (Teledyne Leeman Labs, Hudson, NH, USA). The results are summarized in Table 1.

The X-ray diffraction (XRD) analysis [22] confirmed that the lead cake consists of anglesite (PbSO_4_) as the dominant phase, accompanied by bassanite (CaSO_4_·0.5H_2_O), franklinite (ZnFe_2_O_4_), hematite (Fe_2_O_3_), and a minor amount of sphalerite (ZnS).

Before the experiments, NaCl was dried at 105 °C for 12 h to remove residual moisture and ensure reagent stability. After drying, the NaCl and the lead cake were thoroughly mixed in a specified mass ratio and ground in an agate mortar to obtain a homogeneous fine mixture suitable for subsequent processing.

Chlorination roasting of the lead cake with NaCl was carried out in alumina crucibles, which are chemically inert and resistant to high temperatures. The experiments were conducted at various roasting temperatures using a muffle furnace (model FHP-05—Witeg, Germany). Upon completion of the thermal treatment, the crucibles were removed from the furnace and allowed to cool to room temperature. The resulting roasted products were then ground and subjected to the leaching stage.

Two types of leaching procedures were applied. The first one, referred to as aqueous leaching, represents water leaching without the addition of any reagent. The second one, defined as acid leaching, was performed in a hydrochloric acid solution. In both cases, the leaching experiments were carried out in a vessel fitted with a reflux condenser and placed in a thermostatically controlled water bath, with continuous magnetic stirring to ensure homogeneous suspension of the solids and to prevent solution loss due to evaporation. The leaching time for both aqueous and acid leaching experiments was fixed at 1 h.

Upon completion of the experiments, the resulting pulp was rapidly filtered, and the insoluble residue was dried at 353 K for 24 h and subsequently weighed using an analytical balance KERN, model ABJ 320-4NM. The concentrations of the target metals in the insoluble residues were determined by ICP-OES. The extraction degrees of the target metals were calculated based on the chemical composition of the insoluble residues remaining after the leaching process, as follows:

$$\eta =\left(1-\frac{C_{res}.M_{res}}{C_{cake}.M_{cake}}\right)\cdot 100,\;\%$$

where

*η*—extraction degrees, %;

C_cake_—metal content in the initial lead cake (wt.%);

C_res_—metal content in the leaching residue (wt.%);

M_cake_—mass of the initial sample (g);

M_res_—mass of the residue after leaching (g).

The phase composition of the insoluble residues was determined by XRD analysis using a Philips PW 1050 diffractometer (Philips Analytical, Eindhoven, Netherlands) equipped with Cu-Kα radiation (λ = 1.5406 Å), operating at 40 kV and 30 mA, with data collected over a 2θ range of 5° to 90°.

The morphology and phase composition of the insoluble residues obtained after the combined process were examined using a scanning electron microscope (SEM, Carl Zeiss Microscopy GmbH, Oberkochen, Germany, coupled with Energy-Dispersive X-ray Spectroscopy (EDS, Oxford Instruments, Oxford, UK).

## 3. Results and Discussions

### 3.1. Thermodynamic Assessment

Thermodynamic evaluation was carried out in order to assess the feasibility of converting lead sulfate (PbSO_4_) into lead chloride (PbCl_2_) during chlorination roasting. The standard Gibbs free energy change (ΔG°) was calculated as a function of temperature for the following reactions:

PbSO_4_ + 2NaCl ⟶ PbCl_2_ + Na_2_SO_4_ (1)

PbSO_4_ + 2KCl ⟶ PbCl_2_ + K_2_SO_4_ (2)

PbSO_4_ + CaCl_2_ ⟶ PbCl_2_ + CaSO_4_ (3)

**Figure 1:**
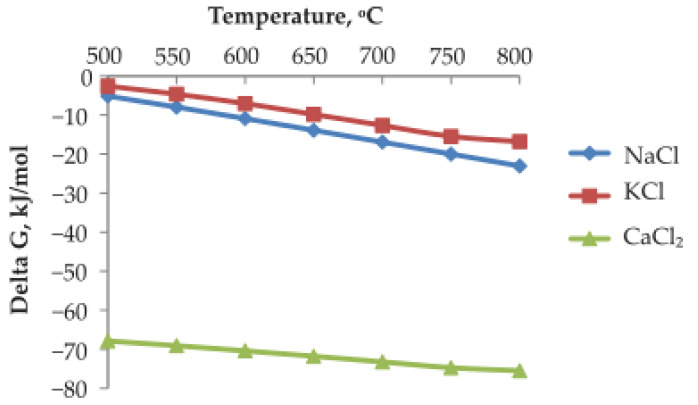
Temperature influence on Gibbs free energy (ΔG).

The calculated ΔG° values for these reactions as a function of temperature are presented in Figure 1. The results show that ΔG° is negative across the investigated temperature range, indicating that the conversion of PbSO_4_ to PbCl_2_ is thermodynamically favorable. Among the chloride reagents considered, NaCl was selected for use in experimental work due to its low cost, wide availability, and ease of handling.

The formation of PbCl_2_ is particularly advantageous for subsequent hydrometallurgical processing because PbCl_2_ exhibits substantially higher solubility in chloride-containing acidic solutions compared to PbSO_4_. Therefore, the thermodynamic analysis confirms that chlorination roasting is an effective activation step that facilitates the dissolution of lead during the leaching stage.

### 3.2. Chlorination Roasting—Aqueous Leaching

Chlorination roasting of the lead cake was performed at three temperatures (500 °C, 600 °C, and 700 °C) and at lead cake-to-NaCl mass ratios of 1:1, 1:2, and 1:3, with roasting durations of 1 h and 2 h. After roasting, the obtained materials were subjected to aqueous leaching at 90 °C for 1 h at a solid-to-liquid ratio of 1:100 under continuous stirring. Water leaching was applied as a reference case to examine the dissolution behavior of Pb- and Ag-phases formed during chlorination roasting.

#### 3.2.1. Effect of Lead Cake-to-NaCl Mass Ratio and Roasting Temperature

**Figure 2:**
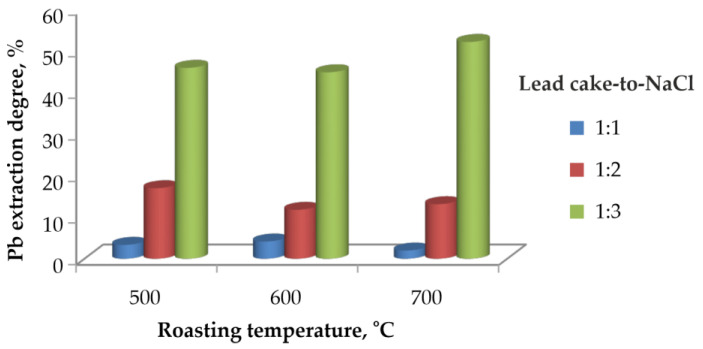
Effect of chlorination roasting temperature and NaCl dosage on the lead extraction degree.

The influence of the lead cake-to-NaCl mass ratio on the leaching degree of lead at different chlorination roasting temperatures is presented in Figure 2.

All experiments were carried out at a roasting duration of two hours. It is clearly observed that increasing the amount of NaCl results in a higher lead extraction degree. At a ratio of 1:1, the extraction degree remains below 10% at all temperatures, indicating insufficient chlorination and incomplete conversion of PbSO_4_ to PbCl_2_. Increasing the ratio to 1:2 results in moderate improvement, with extraction degrees of approximately 10–20%.

The highest extraction degree is achieved at a ratio of 1:3, reaching nearly 50% at 500 °C and 600 °C, and around 55% at 700 °C. However, these extraction degrees are still relatively low under aqueous leaching conditions, which confirms that chlorination roasting alone is insufficient for efficient Pb removal. The slightly higher value observed at 700 °C is possibly associated with partial volatilisation or redistribution of PbCl_2_, resulting in reduced Pb retention in the solid phase rather than enhanced dissolution during aqueous leaching. Therefore, roasting temperatures of 500–600 °C are considered more favourable, as they limit Pb losses to the gas phase.

Thermodynamic calculations for Reaction (1) indicate that significantly less than a 1:1 mass ratio of NaCl to lead cake is theoretically required to achieve complete conversion to PbCl_2_. However, our experimental results clearly demonstrate that under such conditions chlorination proceeds only to a limited extent. This behavior is attributed to the solid-state nature of the reaction, where the diffusion of chloride ions through the PbSO_4_ lattice becomes the rate-controlling step. Therefore, the use of an excess of NaCl improves the reactive contact, overcomes the diffusion constraints, and enables nearly complete chlorination and subsequent dissolution of Pb and Ag.

#### 3.2.2. Effect of Roasting Duration

**Figure 3:**
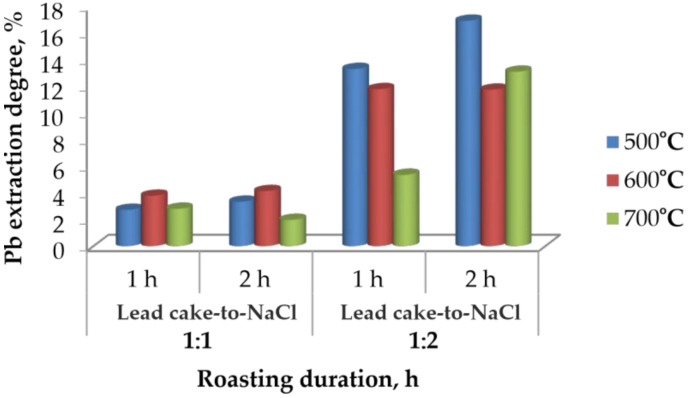
Lead extraction degree as a function of roasting duration at different temperatures and lead cake-to-NaCl ratios.

The effect of roasting duration at different temperatures and lead cake-to-NaCl mass ratios is presented in Figure 3.

At a ratio of 1:1, the extraction degree remains below 10% for both 1 h and 2 h of roasting, regardless of temperature. This confirms that when chloride supply is insufficient, the conversion of PbSO_4_ to PbCl_2_ proceeds only to a very limited extent.

When the ratio is increased to 1:2, the extraction degree of lead improves significantly. The highest values are obtained at 500 °C, while at 600 °C and 700 °C the extraction slightly decreases, most likely due to reduced reactivity of the roasted product at elevated temperature. Extending the roasting duration from 1 h to 2 h results in only a minor increase in extraction, indicating that the chlorination reactions approach completion within the first hour.

Overall, the extraction efficiencies obtained under aqueous leaching conditions remain low and practically unsatisfactory (<50% even at the best parameters). This demonstrates that chlorination roasting followed by water leaching alone cannot ensure effective lead removal.

The chemical composition of the residues obtained after aqueous leaching of the roasted material (roasted at a lead cake-to-NaCl mass ratio of 1:3 and a roasting duration of 2 h) was determined by ICP-OES analysis. The results are presented in Table 2.

As seen from the table, the three insoluble residues obtained after aqueous leaching are characterized by high residual Pb and Ag contents (approximately 17–20% Pb and 0.04–0.05% Ag), indicating that lead and silver were not fully extracted. The limited efficiency of aqueous leaching after chlorination roasting cannot be attributed solely to the high solid-to-liquid ratio (L/S = 1:100). Although dilution reduces the effective chloride concentration, the primary limiting factors are the low intrinsic solubility of PbCl_2_ in neutral aqueous media and the absence of stable Pb–Cl complex formation at near-neutral pH. Under these conditions, PbCl_2_ dissolves only to a limited extent and readily reprecipitates, while AgCl remains practically insoluble. As a result, a substantial fraction of the chlorinated Pb- and Ag-bearing phases remains in the solid residue. In contrast, acidic leaching in HCl promotes both proton-assisted dissolution and the formation of soluble chloride complexes (e.g., PbCl_3_^−^, PbCl_4_^2−^ and AgCl_2_^−^), leading to near-quantitative extraction of Pb and Ag.

To verify this interpretation, an additional experiment was carried out in which the material was roasted with NaCl at 500 °C and a lead cake-to-NaCl mass ratio of 1:3, followed by acid leaching with 1 M HCl at a solid-to-liquid ratio of 1:10 and a temperature of 90 °C. The resulting insoluble residue (Sample 4) contained only 0.96% Pb and 0.010% Ag, clearly demonstrating that acid leaching at a lower solid-to-liquid ratio is significantly more effective for dissolving PbCl_2_ and AgCl.

Furthermore, the behavior of silver generally follows that of lead. During chlorination roasting, Ag is converted predominantly into AgCl, which exhibits low solubility in neutral aqueous media, leading to its enrichment in the solid residue. However, during the acid leaching step, the pronounced decrease in Ag content indicates dissolution of AgCl and its transfer into solution alongside Pb extraction.

### 3.3. XRD Analysis

**Figure 4:**
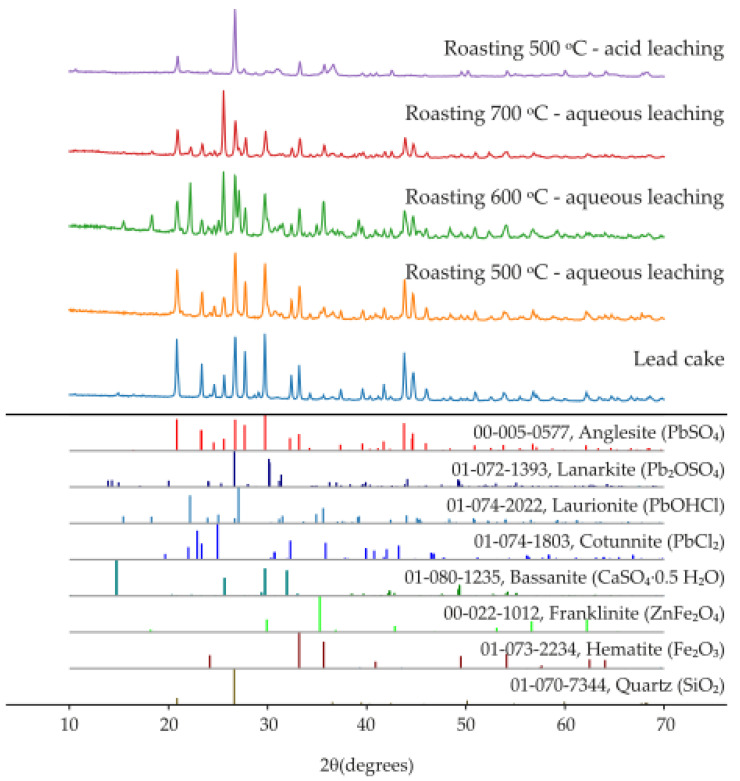
XRD patterns of the initial lead cake and the roasted materials obtained under different roasting conditions.

The insoluble residues obtained after leaching of the roasted materials (Table 2) were analyzed by X-ray diffraction, and the corresponding diffraction patterns are shown in Figure 4.

Comparison with the initial lead cake indicates that chlorination roasting with NaCl leads to the conversion of PbSO_4_ into chloride-containing phases, resulting in the formation of PbCl_2_ and Pb(OH)Cl, as well as partial formation of Pb_2_(SO_4_)O. The obtained results showed that at temperature of 500 °C, chlorination is initiated, but a considerable amount of PbSO_4_ remains unconverted; at 600 °C, PbCl_2_ and Pb(OH)Cl are the dominant phases and the conversion is more complete; while at 700 °C, PbCl_2_ was predominant phase, but partial volatilization occurred, leading to losses of lead.

**Figure 5:**
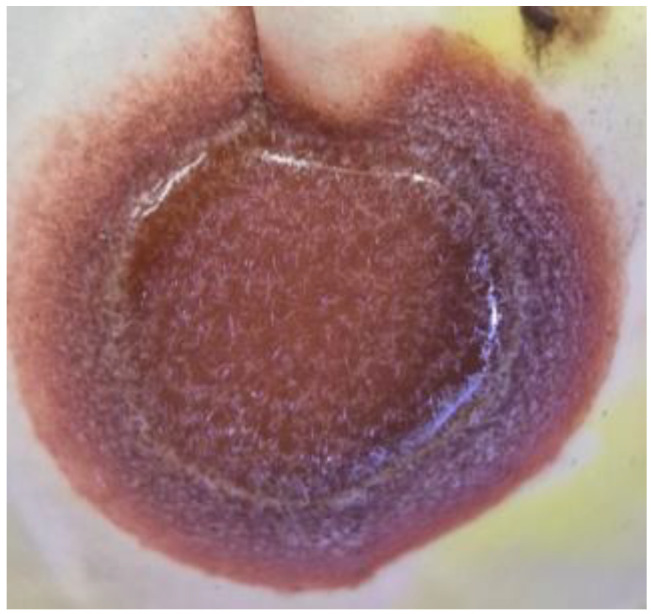
Needle-shaped PbCl_2_ crystals formed during washing of the roasted product.

During washing of the roasted product, the formation of needle-shaped PbCl_2_ crystals was observed (Figure 5), confirming the chlorination pathway. Regardless of the roasting conditions, ZnFe_2_O_4_, Fe_2_O_3_, and SiO_2_ remain stable and constitute the main matrix of the solid residue.

In contrast, acid leaching with 1 M HCl leads to complete dissolution of Pb- and Ag-bearing phases, including sulfates (e.g., PbSO_4_), chlorides (e.g., PbCl_2_, AgCl), and basic chlorides (e.g., Pb(OH)Cl).

After this treatment, the residue consists primarily of SiO_2_, ZnFe_2_O_4_, and Fe_2_O_3_, which are chemically stable under the applied conditions. Therefore, chlorination roasting with NaCl at 500–600 °C is identified as optimal for converting PbSO_4_ into easily leachable chloride phases, whereas roasting at 700 °C leads to Pb loss due to the volatility of PbCl_2_.

### 3.4. SEM/EDS Analysis

The microstructural and energy-dispersive X-ray spectroscopy (EDS) observations complement the XRD results and provide information on the spatial distribution of the phases in the solid residues after roasting and leaching. Two samples were examined by SEM/EDS:

- Sample 1: obtained after chlorination roasting at 500 °C followed by aqueous leaching;
- Sample 4: obtained after chlorination roasting at 500 °C followed by acid leaching with 1 M HCl.

The same roasting temperature of 500 °C and the same lead cake-to-NaCl mass ratio (1:3) were deliberately selected for both samples in order to ensure a direct and reliable comparison of the effect of the leaching medium (aqueous versus acidic) on the microstructure and phase distribution of the residues.

**Figure 6:**
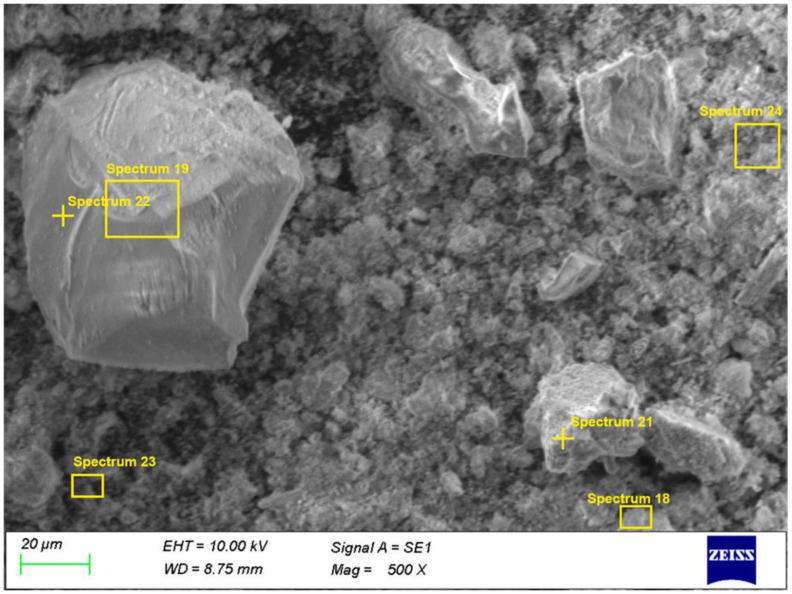
SEM microstructure of Sample 1 (500 °C, aqueous leaching).

In Sample 1, a heterogeneous microstructure is observed, consisting of bright domains corresponding to Pb-bearing phases (e.g., residual PbSO_4_ and newly formed chloride phases), and darker regions related to Zn- and Fe-bearing spinel-type phases (primarily ZnFe_2_O_4_), as shown in Figure 6.

**Figure 7:**
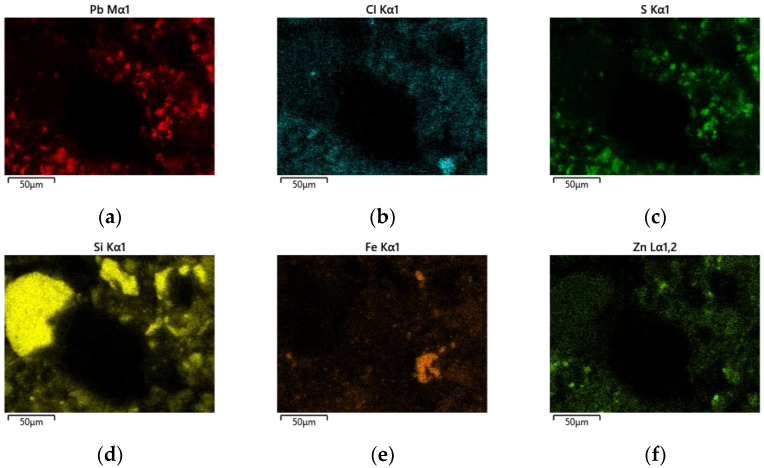
EDS elemental mapping in Sample 1 (500 °C, aqueous leaching). (**a**)—Pb; (**b**)—Cl; (**c**)—S; (**d**)—Si; (**e**)—Fe; (**f**)—Zn.

In addition to point EDS measurements, elemental distribution maps were obtained to visualize the spatial localization of Pb-bearing phases within the solid matrix. The elemental maps for Sample 1 (Figure 7) show heterogeneous distribution of Pb, Cl, and S, occurring as localized enriched regions associated with PbCl_2_, PbSO_4_ and mixed oxychlorosulfate phases. These Pb-rich domains are embedded within a continuous Zn–Fe–Si matrix corresponding to ZnFe_2_O_4_, Fe_2_O_3_ and SiO_2_. The presence of Pb-, Cl-, and S-rich regions confirms incomplete removal of Pb-bearing phases during aqueous extraction.

EDS spectra 18, 21, 23, and 24 (Table 3) show high Pb contents (20–60%), along with variable Cl and S levels, confirming the presence of PbCl_2_, Pb(OH)Cl, PbSO_4_, and Pb_2_(SO_4_)O. Spectrum 21 (59.3% Pb, 7.6% S) is characteristic of residual sulfate phases, while spectrum 18 (25.5% Pb, 3.7% Cl) indicates chlorinated phases. Spectrum 23 reflects partial coverage of ZnFe_2_O_4_ grains by Pb-bearing phases, which suggests incomplete removal of lead during aqueous leaching. In contrast, spectra 19 and 22 show negligible Pb and are dominated by ZnFe_2_O_4_, Fe_2_O_3_, and SiO_2_, forming the stable matrix of the solid residue.

**Figure 8:**
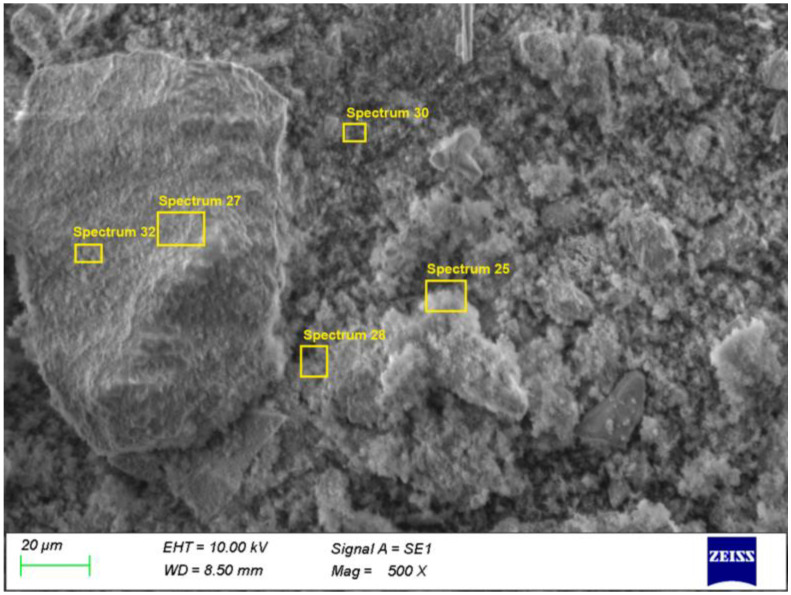
SEM microstructure of Sample 4 (500 °C, acid leaching).

The microstructure of Sample 4 (500 °C, acid leaching) appears more porous and less compact, consistent with the removal of Pb-containing phases (Figure 8).

**Figure 9:**
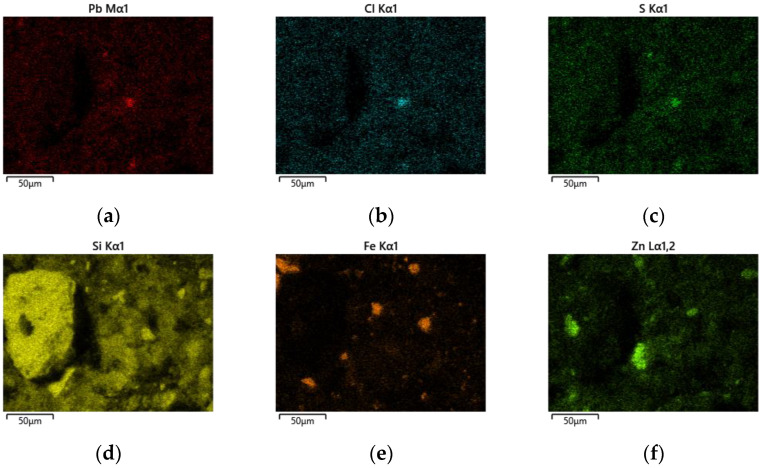
EDS elemental mapping in Sample 4 (500 °C, acid leaching). (**a**)—Pb; (**b**)—Cl; (**c**)—S; (**d**)—Si; (**e**)—Fe; (**f**)—Zn.

The elemental maps for Sample 4 (Figure 9) show the disappearance of Pb-, Cl-, and S-enriched regions, while the Zn–Fe–Si matrix remains structurally preserved, confirming the complete dissolution of Pb-bearing phases during acid leaching with 1 M HCl. These observations are in full agreement with the XRD and ICP-OES results.

In spectrum 25 (Table 4), Pb is present at only 5.5%, while in spectra 27, 28, 30, and 32, Pb levels are below 1%, confirming complete dissolution of Pb-bearing phases during acid leaching. High Si, Zn, and Fe contents reflect the presence of quartz, zinc ferrite, and hematite. Spectrum 28 shows Zn–Al enrichment, characteristic of Al-substituted ferrite, known for high acid resistance.

Therefore, the SEM/EDS analysis confirms the phase evolution sequence established by the XRD results: aqueous leaching removes Pb only partially, whereas acid leaching leads to its complete removal, while the stable Zn–Fe–Si matrix remains preserved.

The EDS analyses of both Sample 1 and Sample 4 show that no silver-containing phases are detected. Silver is present at very low concentrations, and we believe it can only be identified by chemical methods, for example, by ICP analysis.

### 3.5. Chlorination Roasting—Acid Leaching

Since aqueous leaching after chlorination roasting did not provide satisfactory Pb and Ag extraction, acidic leaching was evaluated to better understand the influence of leaching chemistry on metal recovery.

#### 3.5.1. Effect of Roasting Temperature on the Extraction Degree of Metals with 1 M HCl

It is necessary to re-evaluate the effect of roasting temperature because it plays different roles during the various stages of hydrometallurgical leaching. During chlorination roasting, temperature controls the extent of conversion of PbSO_4_ to PbCl_2_ and related chlorinated phases. However, during the subsequent acid leaching stage, roasting temperature influences the solubility and stability of the formed Pb-bearing phases. Therefore, the optimal roasting temperature for maximizing chlorination does not necessarily correspond to the optimal temperature for achieving complete dissolution of lead. For this reason, the roasting temperature effect must be assessed independently as well as in the acid leaching stage.

**Figure 10:**
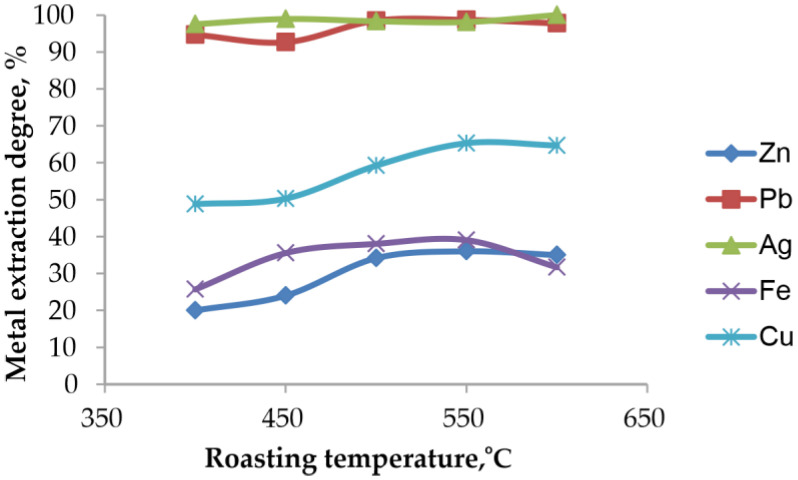
Effect of chlorination roasting temperature on the extraction degree of metals during subsequent acid leaching with 1 M HCl.

The influence of the chlorination roasting temperature on the extraction efficiency of metals during subsequent acid leaching with 1 M HCl after roasting for 1.5 h at a solid-to-liquid ratio of 1:10 and a temperature of 90 °C is presented in Figure 10.

At all roasting temperatures between 400 and 600 °C, the dissolution of Pb and Ag in 1 M HCl exceeds 97%, which reflects the high solubility of the chlorinated phases produced during roasting, including PbSO_4_, PbCl_2_, Pb(OH)Cl, Pb_2_(SO_4_)O and AgCl, in chloride-containing acidic solutions. The dissolution behavior of Ag parallels that of Pb due to the formation of highly soluble Ag–Cl complexes in chloride solutions (primarily AgCl_2_^−^ and AgCl_3_^2−^).

By comparison, the extraction of Zn and Fe remains significantly lower and varies with roasting temperature, which is consistent with the resistance of ZnFe_2_O_4_ and Fe_2_O_3_ to dissolution in acidic chloride solutions. Cu shows a moderate increase in extraction efficiency with temperature, likely reflecting changes in the reactivity of Cu-bearing phases.

Table 5 presents the chemical compositions of the insoluble residues obtained after chlorination roasting followed by acid leaching.

The data show that the residual amounts of lead are very low at all roasting temperatures, with the minimum value of 0.9% Pb at 550 °C. Silver is also present only in trace amounts (0.001–0.003%), confirming its almost complete extraction. The concentrations of Zn, Fe and Cu remain essentially constant, reflecting the stability of the ferrite matrix and indicating that these elements do not dissolve significantly under the applied leaching conditions.

#### 3.5.2. Effect of Pulp Density (Solid-to-Liquid Ratio) at 500 °C

**Figure 11:**
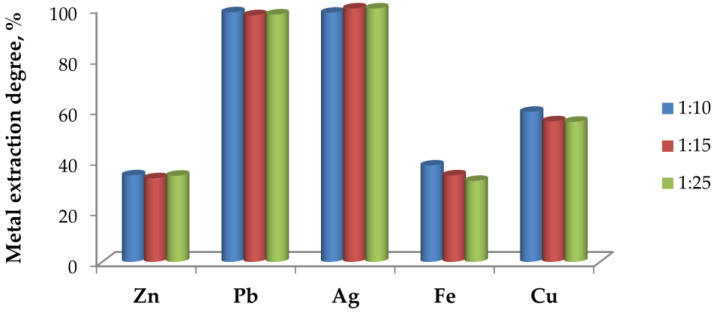
Effect of the solid-to-liquid ratio on the extraction degree of metals.

Figure 11 illustrates the effect of pulp density on metal extraction from the roasted at 500 °C material, followed by leaching with 1 M HCl.

It is evident that changing the solid-to-liquid ratio from 1:10 to 1:25 does not significantly affect the extraction of Pb and Ag; in all cases, the extraction degree remains close to 100%. This confirms the high solubility of Pb chloride phases and the easy dissolution of silver under the applied conditions.

In contrast, Zn and Fe display much lower extraction degrees, while Cu extraction increases at lower solid-to-liquid ratios, with the 1:10 ratio showing the highest dissolution efficiency.

#### 3.5.3. Effect of Acid Concentration

**Figure 12:**
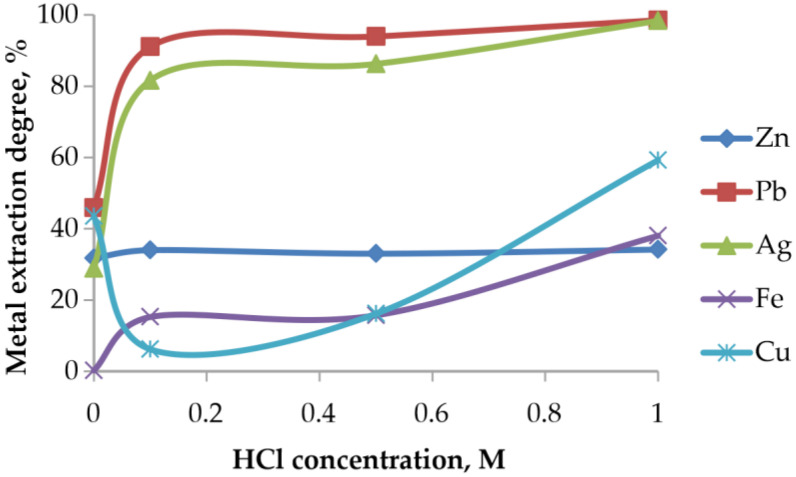
Effect of HCl concentration on the extraction degree of metals.

The increase in HCl concentration has a pronounced effect on the dissolution of Pb and Ag, whereas Zn, Fe, and Cu exhibit different behavior depending on the stability of their host phases (Figure 12).

At low HCl concentrations (0–0.1 M), a sharp increase in Pb and Ag extraction is observed, reflecting the rapid formation of soluble chloride complexes such as PbCl_4_^2−^, PbCl_3_^−^, and AgCl_2_^−^. The leaching degree exceeds 90–95%, indicating that chlorination roasting successfully converted Pb-bearing phases into chloride forms that dissolve readily in chloride media.

In contrast, Zn extraction remains nearly constant (~30%), consistent with the stability of ZnFe_2_O_4_, confirming that Zn remains locked within the ferrite matrix. Fe dissolution increases only at HCl concentrations above 0.6 M, when the protective oxide layers begin to destabilize.

During aqueous leaching, copper undergoes partial dissolution. In the range of 0.1–0.5 M HCl, a reduction in the extraction efficiency is observed, which is attributed to the formation of sparingly soluble phases. However, upon increasing the acid concentration to 1 M HCl, copper enters the solution due to the formation of stable chloro-complex species.

### 3.6. Comparison Between Chlorination Roasting + Acid Leaching and Direct Chloride Leaching

The developed combined process enables efficient and selective recovery of lead from lead cake and results in the formation of a stable residue with low residual Pb content. Chlorination roasting with NaCl at 500–550 °C ensures the activation of PbSO_4_ and its conversion into soluble chloride forms, which are subsequently fully dissolved in 1 M HCl, while the ferrite–silica matrix remains unaffected. The process therefore demonstrates strong potential for integration into industrial flowsheets for the recycling of lead-containing residues. A comparison between the two examined routes—the combined chlorination roasting followed by acid leaching, and direct chloride leaching—clearly shows the advantage of the combined approach both in terms of metal extraction efficiency and the characteristics of the resulting solid residue. After chlorination roasting followed by leaching in 1 M HCl, the mass of the insoluble residue is reduced to 34.20% of the initial material, whereas direct chloride leaching yields a residue corresponding to 45% of the original mass (Table 6). This indicates that the combined process facilitates a greater transfer of metal species into the solution phase, reflecting more complete activation and dissolution of the lead- and silver-bearing phases.

The most notable difference is the significantly lower Pb and Ag concentrations in the residue produced via chlorination roasting followed by acid leaching (0.90% Pb and 0.0027% Ag), compared to the residue obtained by direct chloride leaching (1.57% Pb and 0.0160% Ag).

To clarify the presence of residual Pb in the leaching residue at 90 °C, a thermodynamic assessment of the Pb-Ag-Cl-S-H_2_O system was performed using PHREEQC software (version 3.8.9, LLNL thermodynamic database, llnl.dat) under the experimental leaching conditions (1 M HCl, L/S = 10). The calculations indicate that both PbSO_4_ and PbCl_2_ are thermodynamically undersaturated at 90 °C in chloride-rich acidic media (saturation index, SI < 0; typically SI = −1.4 to −1.08 for PbSO_4_ and SI = −0.26 for PbCl_2_), suggesting that the persistence of Pb in the solid residue is not controlled by equilibrium thermodynamics but rather by kinetic or structural limitations, such as encapsulation of Pb-bearing phases within the roasted matrix or restricted mass transfer during leaching. In this context, chlorination roasting remains beneficial, as it promotes phase transformation, disrupts the solid matrix, and improves the accessibility of Pb- and Ag-bearing phases to the leaching solution, thereby enhancing leaching kinetics and overall extraction efficiency.

A similar interpretation applies to silver, whose enhanced extraction in HCl solution is associated with chloride complex formation, while residual Ag is most likely related to kinetic constraints rather than thermodynamic stability.

In terms of metal extraction, the combined process again shows superior performance. Lead is extracted almost completely (98.68%), and silver to 98.09%, while the values for direct chloride leaching are 96.79% and 84.55%, respectively (Table 7). This improvement results from the conversion of PbSO_4_ into more soluble chloride phases during roasting, which are fully dissolved under acidic leaching conditions.

While Cu also shows improved extraction in the combined process, the extraction of Fe and Zn remains limited in both cases. This behavior is consistent with the persistence of the chemically stable zinc ferrite matrix (ZnFe_2_O_4_), which is resistant to dissolution in both chloride and acidic media. This interpretation is further supported by the XRD and SEM/EDS results, which confirm that after acid dissolution of the roasted material, the solid residue contains mainly ZnFe_2_O_4_, Fe_2_O_3_, and SiO_2_.

### 3.7. Assessment of the Leaching Toxicity of the Final Residue

The leaching behavior of the final solid residue was assessed using the European standard EN 12457-2 [23], which specifies a compliance leaching test at a liquid-to-solid ratio = 10 L/kg under agitation for 24 h. After filtration, the eluate was analyzed for a set of regulated elements by ICP-OES, following the requirements of the Waste Acceptance Criteria (WAC) established in Council Decision 2003/33/EC [24]. The measured concentrations were compared with the limit values for inert, non-hazardous and hazardous waste landfills as presented in Table 8.

The results show that all analysed elements are well below the regulatory limits defined in the WAC. Lead, the key indicator metal, is leached at 0.12 mg/L, significantly lower than the inert waste limit of 0.5 mg/L. Zinc (0.08 mg/L) and copper (0.05 mg/L) are also far below their corresponding thresholds (4 mg/L and 2 mg/L, respectively).

All remaining regulated metals (As, Ba, Cd, Cr, Ni, Se, Hg, Mo, Sb) were below detection limits and therefore compliant with the most stringent WAC category.

These results confirm that the final residue exhibits very low metal mobility and meets the criteria for classification as inert waste, indicating minimal environmental risk upon disposal.

## 4. Conclusions

This study demonstrates that the combined chlorination roasting–acid leaching process is a highly effective approach for the recovery of lead and silver from lead-bearing industrial residues. Chlorination roasting with NaCl at 500–600 °C enables an extensive conversion of PbSO_4_ into easily soluble chloride phases such as PbCl_2_ and Pb(OH)Cl, while preserving the structural stability of ZnFe_2_O_4_, Fe_2_O_3_, and SiO_2_, which form the inert matrix of the final residue.

Aqueous leaching of the roasted product at high solid-to-liquid ratios is insufficient to dissolve the chlorinated phases, leaving up to 20% Pb in the solid residue, which confirms the necessity of acidic extraction following thermal activation. The subsequent acid leaching step of the roasted product with 1 M HCl at a solid-to-liquid ratio of 1:10 plays a decisive role in achieving near-quantitative dissolution of Pb and Ag, reducing the Pb content in the final residue to as low as 0.90% after roasting at 550 °C.

In comparison with single-step chloride leaching, the proposed two-step process delivers higher metal extraction efficiencies and results in a smaller amount of final residue, which is a key factor for sustainable industrial waste management. Furthermore, compliance leaching tests (EN 12457-2) verified that the final residue meets the EU Waste Acceptance Criteria for inert waste, demonstrating a significant reduction in environmental risk.

Overall, the proposed process offers a technically robust and environmentally advantageous solution for the treatment of Pb-containing secondary resources, providing a viable pathway toward improved metal recovery and safer waste disposal.

## Figures and Tables

**Table 1:** Main chemical composition of lead cake, wt.%.

Pb	Ag	Zn	Cu	Fe
26.84	0.0554	1.13	0.17	7.98

**Table 2:** Chemical composition of the residues obtained after aqueous and acid leaching of the roasted material at a lead cake-to-NaCl mass ratio of 1:3.

Sample No.	Process	T, °C	Pb, wt.%	Ag, wt.%	Zn, wt.%	Cu, wt.%	Fe, wt.%
1	Roasting—aqueous leaching	500	19.39	0.0526	1.03	0.13	10.62
2	Roasting—aqueous leaching	600	19.87	0.0458	0.99	0.14	10.67
3	Roasting—aqueous leaching	700	17.28	0.0436	0.96	0.15	10.69
4	Roasting—acid leaching	500	0.96	0.0100	1.76	0.22	8.78

**Table 3:** EDS elemental composition (wt.%) of selected micro-areas in Sample 1.

Element (wt.%)	Sp.18	Sp.19	Sp.21	Sp.22	Sp.23	Sp.24
Pb	25.45	2.93	59.30	0.28	20.62	12.92
Cl	3.69	0.49	0.00	0.04	1.53	0.66
S	0.97	0.00	7.59	0.06	0.64	10.48
O	30.17	57.54	22.87	60.03	34.27	38.34
Si	11.69	38.35	1.08	39.33	11.95	11.31
Fe	12.71	0.63	8.85	0.09	11.62	7.83
Zn	5.58	0.07	0.32	0.17	10.91	3.11
Cu	3.21	–	–	–	–	–
Mn	2.27	–	–	–	1.91	1.12
Al	2.34	–	–	–	5.42	1.74
K	0.77	–	–	–	0.58	0.72
Ca	1.15	–	–	–	0.56	11.79

**Table 4:** EDS elemental composition (wt.%) of selected micro-areas in Sample 4.

Element (wt.%)	Sp.25	Sp.27	Sp.28	Sp.30	Sp.32
Pb	5.49	0.17	0.91	1.11	0.44
Cl	2.25	0.02	0.22	0.23	0.24
S	0.49	0.00	0.05	2.92	0.14
O	44.72	56.93	36.71	52.46	40.82
Si	27.69	39.79	4.86	28.93	15.43
Fe	3.58	0.24	3.43	4.53	3.61
Zn	6.69	1.90	28.40	5.66	19.91
Al	6.01	0.95	22.32	3.26	16.75
Na	2.22	–	3.11	0.91	2.66
K	0.41	–	–	–	–
Ca	0.45	–	–	–	–

**Table 5:** Chemical composition of the insoluble residues after roasting and acid leaching as a function of roasting temperature (wt.%).

Temperature (°C)	Pb	Ag	Zn	Fe	Cu
400	3.13	0.0030	3.99	12.92	0.19
450	4.42	0.0013	3.56	11.48	0.19
500	1.01	0.0023	1.86	12.36	0.17
550	0.90	0.0027	4.46	12.29	0.15
600	1.44	0.0022	4.41	12.92	0.14

**Table 6:** Chemical composition of insoluble residues obtained after chlorination roasting–acid leaching and direct chloride leaching (wt.%).

Process	Residue Mass (% of Cake)	Pb	Cu	Fe	Ag	Zn
Chlorination roasting + acid leaching	34.20	0.90	0.15	12.29	0.0027	4.46
Direct chloride leaching	45.00	1.57	0.19	7.98	0.0160	1.38

**Table 7:** Metal extraction efficiencies (%) obtained via the two leaching routes.

Process	Pb	Ag	Cu	Fe	Zn
Chlorination roasting + acid leaching	98.68	98.09	65.29	39.04	36.01
Direct chloride leaching	96.79	84.55	39.94	45.26	34.63

**Table 8:** Waste Acceptance Criteria (WAC) leaching limits for metals at L/S = 10 L/kg, compared with results from this study, mg/L.

Element	Inert Waste Landfill	Non-Hazardous (Stable, Non-Reactive)	Hazardous Waste Landfill	This Study
As	0.5	2	25	<0.01
Ba	20	100	300	<0.01
Cd	0.04	1	5	<0.01
Cr (total)	0.5	10	70	<0.01
Cu	2	50	100	0.05
Hg	0.01	0.2	2	<0.01
Mo	0.5	10	30	<0.01
Ni	0.4	10	40	<0.01
Pb	0.5	10	50	0.12
Sb	0.06	0.7	5	<0.01
Se	0.1	0.5	7	<0.01
Zn	4	50	200	0.08
Chlorides	800	15,000	25,000	13.5

## Data Availability

The original contributions presented in the study are included in the article. Further inquiries can be directed to the corresponding author.

## Abbreviations

The following abbreviations are used in this manuscript:

SEM	Scanning electron microscope
EDS	Energy Dispersive X-ray Spectroscopy
ICP-OES	Inductively Coupled Plasma Optical Emission Spectroscopy
XRD	X-ray diffraction
SI	Saturation index
WAC	Waste Acceptance Criteria

## References

[1] Fan Z.-Y., Wang L., Zhang R.-Y., Wang Y.-Y., Zhang T.-A., Wang X.-J., Li J.-D., Zhao X.-X., Li W.-J. (2025). Research and application mechanism of chlorination roasting for polymetallic minerals and waste. Miner. Eng..

[2] Li G., Zou X., Cheng H., Geng S., Xiong X., Xu Q., Zhou Z., Lu X. (2020). A novel ammonium chloride roasting approach for the high-efficiency co-sulfation of nickel, cobalt, and copper in polymetallic sulfide minerals. Metall. Mater. Trans. B.

[3] Hu J., Song J., Hu T., Zhang L., Wang Y., Zou F. (2025). Low-temperature chlorination-roasting–acid-leaching uranium process of uranium tailings: Comparison between microwave roasting and conventional roasting. Processes.

[4] Lu C., Yu X., Yang G., Guo Q., Jiao L., Lin J., Deng K., Hu Y. (2025). Mechanistic insights into the chlorination volatilization of oxidized heavy metals via novel staggered chlorination roasting. Waste Dispos. Sustain. Energy.

[5] Li J., Li Y., Gao Y., Zhang Y., Chen Z. (2016). Chlorination roasting of laterite using salt chloride. Int. J. Miner. Process..

[6] Hua Z., Wang J., Wang L., Zhao Z., Li X., Xiao Y., Yang Y. (2014). Selective extraction of rare earth elements from NdFeB scrap by molten chlorides. ACS Sustain. Chem. Eng..

[7] Zhong P., Mu W., Sun W., Zhou Y., Yang R., Wang Q., Lei X., Luo S. (2024). Efficient extraction of Ni, Cu and Co from mixed oxide–sulfide nickel concentrate by sodium chloride roasting: Behavior, mechanism and kinetics. Metall. Mater. Trans. B.

[8] Cui F., Mu W., Wang S., Xin H., Shen H., Xu Q., Zhai Y., Luo S. (2018). Synchronous extractions of nickel, copper, and cobalt by selective chlorinating roasting and water leaching to low-grade nickel–copper matte. Sep. Purif. Technol..

[9] Zhang B.-K., Wang Q.-M., Guo X.-Y., Tian Q.-H. (2023). Mechanism and kinetics for chlorination roasting of copper smelting slag. Trans. Nonferrous Met. Soc. China.

[10] Liu J., Wen S., Chen Y., Liu D., Bai S., Wu D. (2013). Process optimization and reaction mechanism of removing copper from an Fe-rich pyrite cinder using chlorination roasting. J. Iron Steel Res. Int..

[11] Höber L., Witt K., Steinlechner S. (2022). Selective chlorination and extraction of valuable metals from iron precipitation residues. Appl. Sci..

[12] Sinadinovic D., Kamberovic Z., Sutic A. (1997). Leaching kinetics of lead from lead (II) sulphate in aqueous calcium chloride and magnesium chloride solutions. Hydrometallurgy.

[13] Raghavan R., Mohanan P.K., Swarnkar S.R. (2000). Hydrometallurgical processing of lead-bearing materials for the recovery of lead and silver. Hydrometallurgy.

[14] Ruşen A., Sunkar A.S., Topkaya Y.A. (2008). Zinc and lead extraction from Çinkur leach residues by hydrometallurgical methods. Hydrometallurgy.

[15] Farahmand F., Moradkhani D., Safarzadeh M.S., Rashchi F. (2009). Brine leaching of lead-bearing zinc plant residues: Process optimization. Hydrometallurgy.

[16] Behnajady B., Moghaddam J., Behnajady M.A., Rashchi F. (2012). Optimizing lead leaching from zinc plant residues in NaCl–H_2_SO_4_–Ca(OH)_2_ media. Ind. Eng. Chem. Res..

[17] Wang L., Mu W., Shen H., Liu S., Zhai Y. (2015). Leaching of lead from zinc leach residue in acidic CaCl_2_ solution. Int. J. Miner. Metall. Mater..

[18] Xie H., Zhang L., Li H., Koppala S., Yin S., Li S., Yang K., Zhu F. (2019). Efficient recycling of Pb from zinc leaching residues by hydrometallurgy. Mater. Res. Express.

[19] Silwamba M., Ito M., Hiroyoshi N., Tabelin C.B., Hashizume R., Fukushima T., Park I., Jeon S., Igarashi T., Sato T. (2020). Recovery of lead and zinc from zinc plant residues via dissolution–cementation in chloride media. Metals.

[20] Houshmand A.R., Azizi A., Bahri Z. (2024). Recovery of lead from zinc production residue: Process optimization and kinetics. Geosyst. Eng..

[21] Chmielewski T., Gibas K., Borowski K., Adamski Z., Wozniak B., Muszer A. (2017). Chloride leaching of silver and lead from residue after leaching of copper concentrates. Physicochem. Probl. Miner. Process..

[22] Iliev P., Lucheva B., Kazakova N., Stefanova V. (2025). Recovery of iron, silver and lead from zinc ferrite residue. Materials.

[23] (2002). Characterisation of Waste-Leaching-Compliance Test for Leaching of Granular Waste Materials and Sludges—Part 2: One Stage Batch Test at a Liquid to Solid Ratio of 10 L/kg for Materials with Particle Size Below 4 mm.

[24] European Council (2003). Council Decision 2003/33/EC establishing criteria and procedures for the acceptance of waste at landfills. Off. J. Eur. Union.

